# Dynamic Cognitive–Motor Training versus Cognitive Computer-Based Training in People with Multiple Sclerosis: A Preliminary Randomized Controlled Trial with 2-Month Follow-Up

**DOI:** 10.3390/jcm13092664

**Published:** 2024-05-01

**Authors:** Marco Tramontano, Ornella Argento, Nicola Manocchio, Chiara Piacentini, Amaranta Soledad Orejel Bustos, Sara De Angelis, Michela Bossa, Ugo Nocentini

**Affiliations:** 1 Department of Biomedical and Neuromotor Sciences (DIBINEM), Alma Mater Università di Bologna, 40138 Bologna, Italy; 2 Unit of Occupational Medicine, IRCCS Azienda Ospedaliero-Universitaria di Bologna, 40138 Bologna, Italy; 3 Fondazione Santa Lucia IRCCS, 00179 Rome, Italy; o.argento@cbpt.org (O.A.); chiarapiacentini95@gmail.com (C.P.); a.orejel@hsantalucia.it (A.S.O.B.); s.deangelis@hsantalucia.it (S.D.A.); bossa.michela@gmail.com (M.B.); u.nocentini@hsantalucia.it (U.N.); 4 Research Center CBPT, 00196 Rome, Italy; 5 Department of Clinical Sciences and Translational Medicine, University of Rome Tor Vergata, 00133 Rome, Italy; nicola.manocchio@uniroma2.it; 6 Department of Movement, Human and Health Sciences, University of Rome “Foro Italico”, 00135 Rome, Italy

**Keywords:** multiple sclerosis, rehabilitation, dual-task, cognitive impairment, cognitive therapy

## Abstract

**Background:** Recent studies underscore the intricate relationship between cognitive and motor impairments in Multiple Sclerosis (MS), often exacerbated by CNS damage compromising neural connections. These cognitive–motor deficits contribute to reduced efficiency in daily activities and heightened risks of falls and accidents. The combination of challenging cognitive–motor training in a more ecological setting could improve cognitive functions in people with MS (PwMS). **Objective:** This study aims to compare the impact of dynamic cognitive–motor training versus computer-based cognitive training on overall cognitive efficiency in PwMS. **Methods:** Thirty-eight PwMS were recruited through the neurorehabilitation services of an Institute of research and health. Twenty-four participants were randomly assigned to the Cognitive-Motor group (CMg) and Cognitive Therapy group (CTg). Participants underwent three training sessions per week for four weeks, each lasting 50 min. The primary outcome was a comprehensive cognitive assessment using the Cognitive Impairment Index (CII), and the secondary outcomes were the Multiple Sclerosis Quality of Life Questionnaire MSQOL-54 and the Stroop Color Word Interference Test (SCWT). **Results:** Significant differences in the CII scores across T0, T1, and T2, as indicated by Friedman’s test (χ2(2) = 14.558, *p* = .001), were found in the CMg. A significant difference in the change in health subscale of the MSQOL-54 was observed when comparing the groups across T0, T1, and T2 (χ2(2) = 6.059, *p* = .048). There were also statistically significant differences for the emotional well-being (χ2(2) = 7.581, *p* = .023) and health distress (χ2(2) = 11.902, *p* = .003) subscales. Post hoc analysis showed a statistically significant improvement in health-related quality of life (HRQOL) for the former at T1 vs. T0 (Z = −2.502, *p* = .012 and for the latter at T2 vs. T0 (Z = −2.670, *p* = .008), respectively. **Conclusions:** Our results support the combination of cognitive–motor training to enhance cognitive functional outcomes and quality of life compared to computer-based cognitive training in PwMS.

## 1. Introduction

Multiple Sclerosis (MS) is an inflammatory, demyelinating, and degenerative disease of the Central Nervous System (CNS), with a chronic long-lasting course, and is one of the leading causes of disability among young and middle-aged adults [1]. Symptoms of MS vary widely among individuals, encompassing motor and sensory impairments, cognitive deficits, fatigue, and autonomic dysfunction. Recent studies underscore the intricate relationship between cognitive and motor impairments in MS, often exacerbated by CNS damage compromising neural connections [2]. This cognitive–motor deficits interplay contributes to reduced efficiency in daily activities and heightened risks of falls and accidents [3,4]. Indeed, cognitive impairment (CI) affects vital aspects of daily functioning in people with MS (PwMS) such as information processing speed, attention, memory, and executive functions [5]. In addition to the factors mentioned, cognitive and daily functioning in PwMS can also be influenced by mood, personality traits, and fatigue. Research suggests that behavioral rigidity and extroversion are particularly associated with fatigue, depressive symptoms, and a lower quality of life. Those exhibiting these traits often report higher levels of cognitive fatigue, concentration difficulties, and learning impairments. Conversely, physical fatigue tends to be linked with lower extroversion, possibly because more extroverted individuals engage more in social and physical activities, which can enhance motor performance. Notably, physical training has been found to alleviate cognitive impairment and fatigue in PwMS [6,7,8].

While pharmacological interventions have shown limited efficacy in addressing cognitive deficits, rehabilitation strategies offer promising avenues for improvement [9,10,11,12]. The traditional therapeutic approach that favors the separate use of motor training and cognitive rehabilitation may not truly reflect the limitations that PwMS have to face during the activities of daily living, in which they are very often called upon to carry out a dual-task (DT). Furthermore, functional and goal-oriented locomotion is not purely automatic; instead, it requires the involvement of higher-level cognitive processes, highlighting the strong relationship existing between cognitive functioning and walking [13]. Indeed, cognitive–motor interventions have emerged as a compelling approach, targeting not only the motor but also the emotional dimensions of MS [14]. Several studies have already suggested that integrated cognitive–motor rehabilitation programs promote neuroplasticity, facilitating functional gains in both motor and cognitive domains [15,16,17]. Task-specific training holds the potential for skill transfer to untrained functions, fostering re-learning and functional recovery [16]. Given the potential of integrated rehabilitation to mitigate cognitive–motor interference (CMI) and enhance quality of life, there is a need for research evaluating the efficacy of cognitive–motor training compared to isolated cognitive interventions. We hypothesize that the combination of challenging cognitive–motor training in a more ecological setting could improve cognitive functions in PwMS. For these reasons, this study aims to compare the impact of dynamic cognitive–motor training versus computer-based cognitive training on overall cognitive efficiency in PwMS.

## 2. Materials and Methods

### 2.1. Study Design

This study comprised a two-arm, parallel, assessor-blinded Randomized Controlled Trial (RCT) with 1:1 allocation, which was approved by the Local Ethics Committee of the “Santa Lucia Foundation” (FSL) Institute for Research and Health Care, with protocol number CE/PROG.812 obtained prior to enrolling the first participants. The study sample was randomly divided into two groups: cognitive–motor therapy (CMg) and cognitive therapy (CTg). A neurologist not involved in the evaluation protocol assessed the patients’ eligibility to participate based on the inclusion and exclusion criteria. Participants underwent assessments by experienced psychologists and physiotherapists at three time points: before starting the training (T0), at the end of the training (T1), and 2 months after T1 (T2). This study adhered to the CONSORT (S1) and TIDieR checklists (S2).

### 2.2. Participants

Between December 2020 and July 2023, participants were recruited through the neurorehabilitation services of the FSL. Eligibility criteria included age of 18 years or older; MS diagnosis (relapsing–remitting; RR or secondary progressive; SP) according to the revised McDonald criteria [18]; mild to moderate difficulty in mobility with an Expanded Disability Status Scale (EDSS) score of 1.5–6.0; ability to walk independently for at least 50 m even with the use of an aid (cane or walker); no exacerbation in the past 4 weeks. Exclusion criteria included untreated psychiatric and neurological disorders (other than MS); other clinically significant disorders that interfere with motor or cognitive tasks; steroid therapy within 4 weeks prior to enrollment; significant sensory organ impairments (such as visual or hearing impairments) that interfere with motor or cognitive tasks; occurrence of a lower limb fracture in the three months prior to enrollment. Neuropsychological assessments were performed by two neuropsychologists with extensive experience in cognitive impairment and subsequent disabilities evaluation. Motor assessments and treatment were performed by two physical therapists with at least 5 years of experience in neurorehabilitation.

### 2.3. Randomization and Concealment

Following the baseline assessment, participants were randomly assigned to either the intervention or control groups through a central computer-based randomization system. This system employed permuted block randomization utilizing computer-generated random numbers to ensure balanced group sizes. Allocation concealment was maintained by staff responsible for the reassessments and outcome collection. Enrolment and intervention assignments were conducted by staff not involved in data collection.

Each participant was allocated in the intervention or control group based on the randomization list by assigning a unique identifying code to guarantee data pseudonymization during data collection, analysis, and interpretation processes.

### 2.4. Intervention

Participants underwent three training sessions per week for four weeks, each lasting 50 min. Every session started with 30 min of conventional neuromotor therapy, in addition to which the CMg received 20 min of cognitive–motor therapy and the CTg received computer-based cognitive therapy.

Conventional neuromotor rehabilitation involved various techniques such as muscle stretching, mobilizations, gait training, and balance exercises on unstable surfaces [19].

In the combined cognitive–motor therapy, participants engaged in a dual-task paradigm where they responded to unpredictable auditory stimuli by rotating their heads toward the sound while identifying visual targets. These tasks were performed while walking on unstable surfaces and treadmills [20,21].

Cognitive rehabilitation therapy focused on attention and executive functions using Rehacom software. One module involved memorizing and identifying target stimuli among similar ones, while another simulated driving, requiring the patient to respond to various road signs and sound cues. Task difficulty was adjusted based on performance to prevent frustration, each module lasting 10 min.

### 2.5. Primary Outcome

The primary outcome was a comprehensive cognitive assessment using the validated Italian version of the Minimal Assessment of Cognitive Function in MS (MACFIMS; [22,23]). This battery consisted of the California Verbal Learning Test-II (measurement of verbal learning and memory), Brief Visuospatial Memory Test—Revised (visuospatial memory test), Symbol Digit Modalities Test (information processing speed; SDMT; oral version), Benton Judgment of Line Orientation test (measures the accuracy of spatial orientation judgments), Controlled Oral Word Association Test (measure of phonemic fluency), and Delis–Kaplan Executive Function System Sorting Test, Paced Auditory Serial Addition Test in 3 and 2 s versions (measure of working memory). The battery administration provides a Cognitive Impairment Index (CII) that indicates the overall cognitive efficiency across different degrees of CI in PwMS. Higher CII scores indicates greater impairment.

### 2.6. Secondary Outcomes

We considered two secondary outcome measures. The Multiple Sclerosis Quality of Life Questionnaire MSQOL-54, an MS-specific quality of life questionnaire composed of a core measure, the 36-item Short-Form health survey (SF-36), and 18 additional items exploring domains relevant to patients with MS (MS-18 module). The total 54 items are divided into 14 subscales: Physical Health, Role limitations due to physical problems, Role limitations due to emotional problems, Pain, Emotional well-being, Energy, Health Perceptions, Social Function, Cognitive Function, Health distress, Sexual Function, Change in health, Satisfaction with sexual function, Overall quality of life [24]. The other secondary outcome was the Stroop Color Word Interference Test (SCWIT) [25,26], a widely employed neuropsychological assessment tool utilized to measure the capacity for inhibiting cognitive interference. This interference arises when the processing of one stimulus feature hampers the simultaneous processing of another stimulus attribute, a phenomenon recognized as the Stroop Effect affecting the performance in terms of errors (SCS I-E) and time (SCS I-T).

### 2.7. Adverse Events

Adverse events encompassed falls or injuries associated with the interventions or intervention equipment. Participants were urged to promptly report any such incidents, which were additionally tracked through monthly calendars and telephone check-ins.

### 2.8. Sample Size

A priori analysis of sample size was conducted using G*Power Version 3.1.9.4 software. Considering two groups and three repeated assessments, an effect size of 0.6, a type I error probability of 0.05, and a power effect of 0.80, the minimum required group size was 24 PwMS, accounting for dropouts (10%).

### 2.9. Statistical Analysis

Statistical analysis was carried out using SPSS (v.23, IBM Corp, Armonk, NY, USA). The normality of data distribution was assessed through the Shapiro–Wilk test (*p* > 0.05). Data were assessed for multivariate outliers using a Mahalanobis Distance Test [27]. Nonparametric analyses were performed using the independent Mann–Whitney U test between the two groups. The Friedman test was used for the within-subjects comparison of the two groups at times T1–T0, T2–T0, and T2–T1. Post hoc analysis with Wilcoxon signed-rank tests was conducted with a Bonferroni correction applied, resulting in a significance level set at *p* < 0.017.

## 3. Results

**Figure 1:**
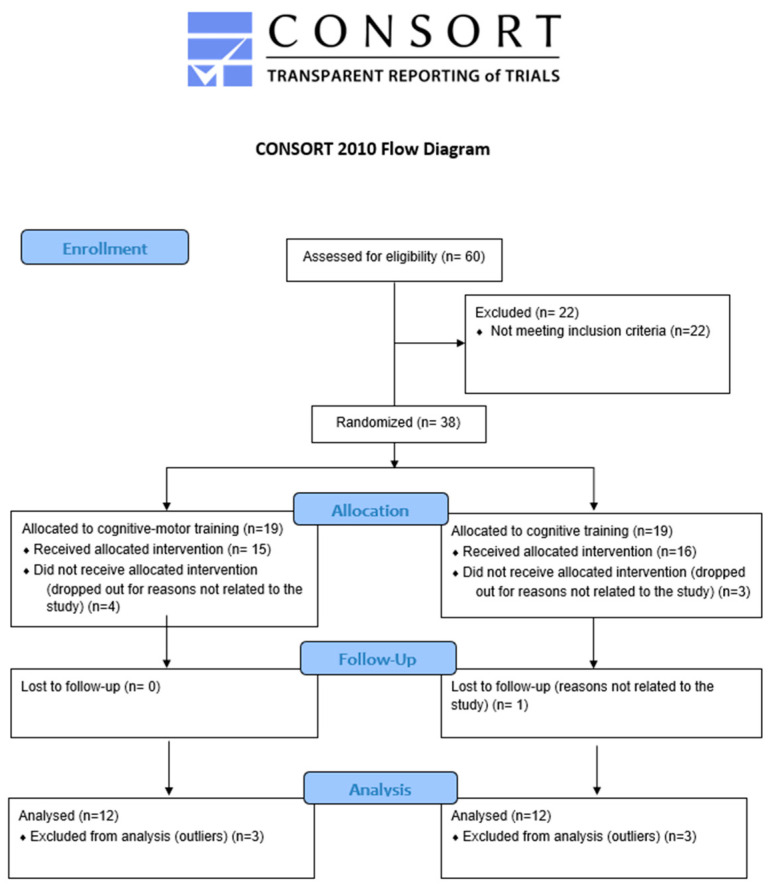
Study design.

Thirty-eight participants were recruited; eight of them dropped out during the treatment period for reasons not related to the study, and six multivariate outliers were identified and removed. Statistical analysis was performed using the data of 24 PwMS, after completion of the three evaluations (CMg = 12, TCg = 12). Demographic and clinical characteristics are reported in Table 1 (Figure 1).

**Figure 2:**
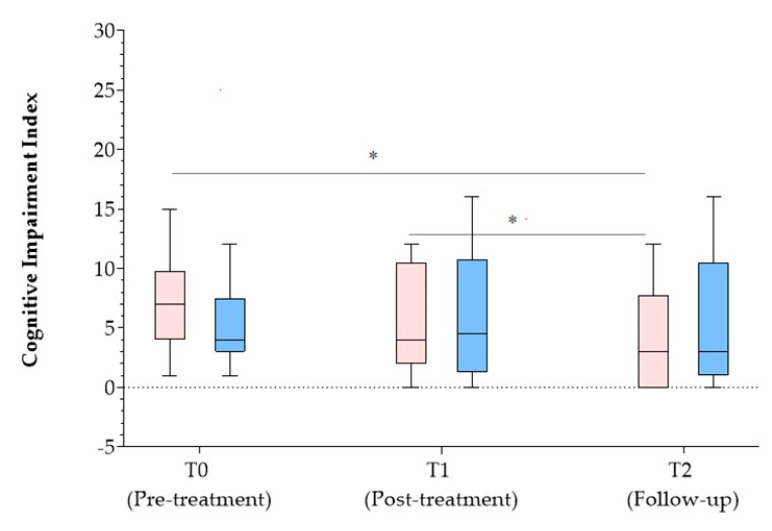
Cognitive Impairment Index score for both CM and CT groups. Pink box plot: Cognitive–Motor group; Blue box plot: Cognitive therapy group; * *p* < 0.05.

**Figure 3:**
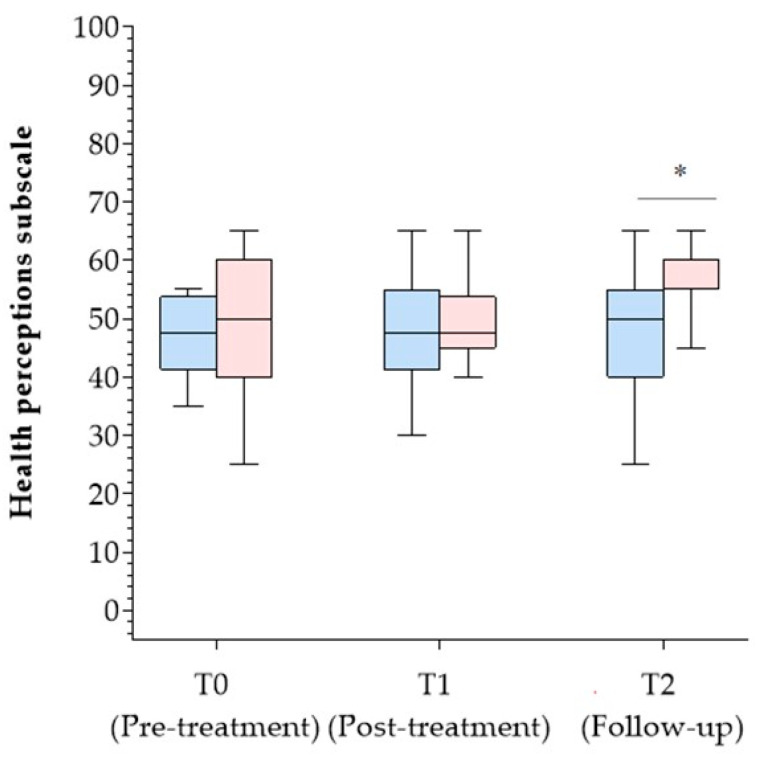
Comparison in the between-group analysis for the *health perceptions* subscale MSQOL-54. Pink box plot: Cognitive–Motor group; Blue box plot: Cognitive therapy group; * *p* < 0.05.

Significant differences in the CII scores were found in the within-subjects analysis across T0, T1, and T2. Specifically, a statistically significant difference was observed between T2 and T1 (Z = −2.692, *p* = .007), as well as between T2 and T0 (Z = −2.881, *p* = .004) as reported in Figure 2. The between-subject analysis showed a significant difference in the *health perceptions* subscale of the MSQOL-54 when comparing the groups at T2 (χ2(2) = 6.49, *p* = .039). Specifically, the Mann–Whitney test revealed that the *health perceptions* subscale scores were higher for patients of the CMg (mean rank = 13.63) when compared to CTg (mean rank = 11.38, U = 26.50, z = −2.71, *p* = .007, r = .55) as reported in Figure 3. 

In the CMg, statistically significant differences were found for the *change in health* at T2 vs. T0 (Z = −2.203, *p* = .028) and *health perception* at T2 vs. T1 (χ2(2) = 11.902, *p* = .003) subscales, even though, after post hoc analysis, no significant differences were observed (*p* > .017). The CTg showed significant results for SCS I-E and the *emotional wellbeing* at T1 vs. T0 (χ2(2) = 7.581, *p* = .023) and *health distress* (χ2(2) = 11.902, *p* = .003) subscales of the MSQOL-54 at T1 vs. T0. Specifically, post hoc analysis showed statistically significant changes for *emotional wellbeing* at T1 vs. T0 (Z = −2.502, *p* = .012) and for *health distress* at T2 vs. T0 (Z = −2.670, *p* = .008). However, for the SCS I-E scores, no significant changes were observed among the three assessments (*p* > .017). Comparison of the clinical scale scores in the within-subjects analysis can be found in Table 2. 

No adverse events were observed.

## 4. Discussion

This study aimed to compare the effects of a dynamic cognitive–motor dual-task program with cognitive computer-based training on overall cognitive efficiency. Our results revealed that participants in the CMg demonstrated improvements in the CII and MSQOL-54 subscales when analyzed longitudinally within subjects. Specifically, the CMg exhibited a gradual increase in CII, reaching its peak at T2 compared to T1 and T0. The observed significant benefits among individuals with MS who underwent dynamic cognitive–motor rehabilitation are consistent with the previous literature [28,29,30]. Motor interventions, particularly those emphasizing postural stability and balance, can positively influence cognitive functioning, likely due to the involvement of the cerebellum in planning, motor learning, and various cognitive domains [31,32].

Moreover, the Prioritization Theory [33] offers another plausible explanation for the observed CMI within the DT paradigm. The former posits that the DT effect arises from the limitation that only one information processing operation can occur at a time [34], while the latter argues that although multiple tasks can be executed in parallel, there is a constraint on central processing capacity. The results indicate that when resources are contested, individuals must determine which task to prioritize [33]. Hence, if someone with MS needs to allocate more attention to maintaining posture and stability to reduce risks, cognitive performance may suffer. Reflecting on our findings, the cognitive–motor dual-task training, by improving motor skills, enables participants to allocate greater resources to cognitive tasks. Another consideration is that individuals in the CMg exhibited a more pronounced increase in CII during the follow-up period. This duration might suggest that in the weeks after the training, participants continued to adapt their resource allocation, progressively enhancing cognitive performance. Additionally, this could be attributed to the time necessary for brain plasticity to produce its effects and become clinically noticeable.

Concerning the health perception evaluated through MSQOL-54, the CMg group showed a statistically significant improvement on the *health perceptions* subscale after the 2 months’ follow-up period when compared to the CTg group. As previously noted by Castelli and colleagues [35], there exists a correlation between dual-task performance and subscales of the MSQOL-54. Indeed, diminished motor abilities have a negative impact on both the QoL and work efficiency in PwMS [36,37]. The CTg demonstrated improved performance on the Symbol Digit Modalities Test, characterized by a decrease in errors. Several studies [38,39], conducted with larger participant cohorts, have investigated the effectiveness of computerized cognitive rehabilitation in PwMS, revealing positive outcomes, particularly in the SDMT. Cognitive and emotional deficits hold significant importance for PwMS, given their association with daily activities, reduced social engagement, and poorer health-related quality of life [40,41].

The benefits of cognitive rehabilitation appear to manifest in terms of enhanced self-reported aspects of QoL. Indeed, within our sample, the CTg demonstrated improved emotional well-being, marked by decreased perceived distress related to their health status during follow-up (as indicated by emotional well-being and health distress MSQOL-54 subscales). Emotional well-being may not be directly impacted by cognitive training; however, authors have suggested a potential indirect improvement linked to the generalization of enhanced cognitive function [42]. Previous studies have highlighted a correlation between cognitive functioning and HRQoL, with lower HRQoL scores associated with lower mood and more severe cognitive impairment, indicating that impaired mental health functioning leads to a decline in patients’ QoL [43]. The feasibility and effectiveness of computer-based training for PwMS are well established. Our findings align with previous reports regarding the neuropsychological benefits of such training [44]. Looking ahead, the ability to deliver rehabilitation via computer appears promising in terms of reducing access barriers for individuals with disabilities and facilitating easily executable protocols. From a research standpoint, these protocols are highly reproducible, thus allowing thorough assessment by the international scientific community [45]. To the best of our knowledge, this study represents the first investigation conducted on PwMS comparing the effects of cognitive–motor therapy with cognitive computer-based training. Our findings provide support for the integration of DT therapy within conventional neuromotor therapy, aiming to enhance not only motor abilities but also cognitive functions in PwMS. An innovative aspect of our protocol is the incorporation of unpredictability into the motor task, necessitating attention to postural stability, while the cognitive task is designed to be more ecologically valid, simulating real-life scenarios where attention must be directed to recognize stimuli without requiring complex responses (similar to situations encountered when crossing the street and responding to auditory or visual cues). Our goal with this training was to enhance attentional capacity to effectively integrate the two concurrent operations. Future studies should explore the optimal timing for initiating cognitive–motor training, as well as determine the specific timing and intensity of cognitive–motor therapy. Additionally, incorporating more objective neurophysiological measurements could provide further insights into the mechanisms underlying the observed improvements.

### Limitations

Several limitations should be considered when interpreting our results. Firstly, the sample size was notably limited, primarily due to difficulties in patient recruitment exacerbated by the pandemic, and we also removed six patients from the dataset according to the outlier’s multivariate analysis. Consequently, this limitation may have impacted the outcomes of the post hoc analysis. Furthermore, we did not assess the effect of the MS phenotype (i.e., relapsing–remitting vs. secondary progressive) or any potential differences between early and late disease stages. These factors could have influenced the efficacy and generalizability of our findings.

## 5. Conclusions

Integrating cognitive–motor training may lead to improved cognitive functional outcomes and quality of life compared to computer-based cognitive training alone in PwMS. Complementary neurorehabilitation strategies that prioritize cognitive–motor dual-tasking should be incorporated into conventional training programs to mitigate cognitive–motor interference and enhance overall rehabilitation outcomes.

## Figures and Tables

**Table 1:** Demographic characteristics of the analyzed dataset (n = 24 participants).

	CMg (n= 12)	CTg (n = 12)	Differences between Group
Women (%)	11 (91.7)	7 (58.3)	.181
Age, years SD	48.92 ± 10.13	46.58 ± 11.13	.408
Years since diagnosis, SD	12.08 ± 8.58	12.00 ± 8.71	.981
EDSS mean SD			
Pre-treatment	3.92 ± 1.4	4.25 ± 1.83	.311
Post-treatment	3.92 ± 1.55	4.13 ± 1.73	.379
Follow-up	3.92 ± 1.68	4.13 ± 1.77	.385
Mean Education, years SD	16.75 ± 5.73	14.00 ± 3.19	.160

CMg: Cognitive–Motor group; CTg: Cognitive-Training group; SD: Standard deviation.

**Table 2:** Comparison of the clinical scale scores in the within-subjects analysis.

*Scale Score* *Mean ± SD*		*Group*	*T0*	*T1*	*T2*
*CII*		CMg	7.00 3.91	6.08 5.90 *	3.83 4.63 *
		CTg	5.5 6.14	6.67 6.76	6.25 7.26
*SCS I-E*		CMg	1.30 1.19	0.81 0.83	1.00 1.07
		CTg	1.02 1.01	1.79 2.25	.60 0.83
*MSQOL-54*	Emotional Wellbeing	CMg	53.33 24.32	55.33 25.17 *	54.67 21.66
		TCg	60.33 16.22	70.67 11.36 *	64.00 15.91
	Health Perceptions	CMg	49.58 13.05	48.75 7.11	56.67 4.92 *
		TCg	46.67 7.18	48.33 9.61	46.25 11.51
	Health Distress	CMg	60.83 28.03	60.83 23.24	66.25 26.21 *
		TCg	62.08 18.64	76.67 10.30 *	76.67 14.82
	Change in Health	CMg	31.25 37.12	45.83 38.19	56.25 30.39 *
		TCg	45.83 23.44	54.17 23.44	50.00 30.15

CII: Cognitive Impairment Index; SCS I-E: Stroop Correct Score-Interference Errors; MSQOL-54: Multiple Sclerosis Quality of Life Questionnaire; *: *p* value < 0.05.

## Data Availability

Data are available upon reasonable request to the corresponding author.

## Supplementary Materials

The following supporting information can be downloaded at: https://www.mdpi.com/article/10.3390/jcm13092664/s1, S1 Consort checklist; S2 Tidier checklist.
